# Fabrication and Evaluation of Embroidery-Based Electrode for EMG Smart Wear Using Moss Stitch Technique

**DOI:** 10.3390/s23219012

**Published:** 2023-11-06

**Authors:** Soohyeon Rho, Hyelim Kim, Daeyoung Lim, Wonyoung Jeong

**Affiliations:** 1 Material and Component Convergence R&D Department, Korea Institute of Industrial Technology (KITECH), Ansan 15588, Republic of Korea; rhosh615@kitech.re.kr (S.R.); hyelim1221@kitech.re.kr (H.K.); zoro1967@kitech.re.kr (D.L.); 2 Department of Nano Science and Technology, Sungkyunkwan University, Suwon 16419, Republic of Korea

**Keywords:** textile electrode, embroidery, electromyography, sEMG electrode, smart clothing

## Abstract

Wearable 2.0 research has been conducted on the manufacture of smart fitness wear that collects bio-signals through the wearing of a textile-based electrode. Among them, the electromyography (EMG) suit measures the electrical signals generated by the muscles to check their activity, such as contraction and relaxation. General gel-type electrodes have been reported to cause skin diseases due to an uncomfortable feel and skin irritation when attached to the skin for a long time. Dry electrodes of various materials are being developed to solve this problem. Previous research has reported EMG detectio performance and conducted economic comparisons according to the size and shape of the embroidery electrode. On the other hand, these embroidery electrodes still have foreign body sensations. In this study, a moss sEMG electrode was produced with various shapes (W3 and WF) and loop lengths (1–5 mm). The optimized conditions of the embroidery-based electrodes were derived and analyzed with the tactile comfort factors and sensing performances. As the loop length of the electrode increased, MIU and Qmax increased, but the SMD decreased due to the free movement of the threads constituting the loop. Impedance and sEMG detection performance showed different trends depending on the electrode type.

## 1. Introduction

Textiles perform two primary functions: protecting the body from the environment and aesthetics. On the other hand, people now want smart functions, such as sensing or responding to external stimuli through textiles, due to a paradigm shift in recent years. This interest has led to a new field called e-textiles, which combines textile engineering and electronics; additionally, textile-based wearable sensing technology is an important part of the IoT [1,2,3,4,5]. Many wearable devices are on the market, such as smartwatches and wristbands that detect heart rate, steps, exercise duration, and fitness level. These can be attached to the human body to monitor vital signs but are difficult to personalize, rigid, and difficult to wear on multiple sites. On the other hand, smart clothing is flexible and comfortable to wear. Moreover, it does not require expensive work to modify the design according to the age or gender of the user, and various options for sensor placement are available.

Recently, many studies have reported the manufacture of smart wear capable of measuring bio-signals (heart rate, respiration rate, and movement) [6]. Among them, EMG electrodes that can measure muscle activity have been used for training to improve the rehabilitation effect of professional athletes or prevent injuries [7]. In addition, with the increase in interest in home fitness and home training after COVID-19, many studies are being conducted to develop EMG suits for the general public (non-professional exercisers). Early electrodes for measuring EMG signals (iEMG; intramuscular EMG) could measure muscle microelectricity with high accuracy and low noise because a needle or fine wire electrodes penetrated the muscle. On the other hand, a professional measurer was required when measuring with an electrode directly inserted into the muscle because it could cause injury to the muscle or surrounding skin tissue and even lead to bleeding [8,9]. sEMG (surface electromyography), a non-invasive evaluation method, is conducted by attaching electrodes to the skin surface. Hydrogel (Ag/AgCl) electrodes are generally unsuitable as monitoring electrodes in cases that require long-term use because of problems, such as one-time use, foreign body sensation, and skin disease caused by skin irritation. The electrodes for smart clothing must be skin-friendly and non-irritating, but they must deliver signals well even if the body is moving during long-term monitoring of electromyography. Textile-based electrodes have the advantages of being soft, flexible, and air-permeable [10,11].

Generally, there are three ways to provide textiles with electronic functions by integrating electronic components and circuits: textile-adapted methods, textile-integrated methods, and textile-based methods. Early developers integrated small-sized electronic components into clothing easily and simply [5]. Various clothing manufacturing processes have been applied to produce e-textiles [12,13,14,15,16,17]. Embroidery is the most frequently used technique in the field of e-textiles. The advantages of embroidery technology are the dimensional stability of the designed structure and the excellent reproducibility of the manufacturing process. In addition, embroidered electrodes also have the advantages of softness, flexibility, multi-use, and self-administration without medical assistance. This method of manufacturing electronic textiles using embroidery is called electronic embroidery or e-broidery. Lock stitch and moss stitch techniques are generally used in electronic textile manufacturing. The basic embroidery technique, lock stitch, has a two-dimensional stitch form in which the substrate fabric and embroidery thread are fixed horizontally. On the other hand, the moss stitch has a three-dimensional shape with loops made of threads perpendicular to the substrate fabric. The loop-type moss stitch can move more flexibly than the lock stitch, in which the stitch is in close contact with the fabric [18].

Previous studies have reported the sEMG collection performance and provided economic comparisons according to the size and shape of the embroidery electrodes [14,15,16,17]; however, the electrodes still felt rough to the touch. In this study, sEMG electrodes were fabricated with a more flexible and free moss stitch to improve the adhesion between the electrode and the skin and the comfort of the human body. The electrical performance and EMG collection performance were evaluated. In addition, the adhesion between the body and electrodes and the EMG collection performance were compared and analyzed by evaluating clothing pressure.

## 2. Experimental

### 2.1. Materials

Silver-coated 100% polyamide core and polyester hybrid conductive thread (Silver-tech 120, AMANN group, Bönnigheim, Germany), which was used as a textile electrode sensor in a previous study [17], was also used to fabricate the embroidered moss electrode. The conductive thread has 27.8 tex and 530 Ω/m. The substrate fabric had an elastic composed of 88% polyester and 12% spandex and a weight and thickness of 590 g/m^2^ and 0.90 mm, respectively. The embroidery design of the electrodes was produced using an embroidery design program (EPC_win, ZSK Stickmaschinen GmbH, Krefeld, Germany) and fabricated by a technical embroidery machine (SGVA 0109-825, ZSK Stickmaschinen GmbH, Krefeld, Germany) which has three types of head for different stitching. Among them, the K-head was selected for the moss stitch with protruding loops on the surface.

### 2.2. Embroidery Design for sEMG Moss Electrodes

Table 1 lists the sample code of moss electrodes and their embroidery design factors. Based on a previous study, the diameter of the textile-based electrode was designed and manufactured to be 20 mm. In addition, the shape of the electrodes and the W3 (wave three lines) and WF (wave fill) designs were chosen in a previous study [16]. The textile-type electrodes were designed and embroidered with two shapes (WF and W3) and five loop lengths (1 mm (L1), 2 mm (L2), 3 mm (L3), 4 mm (L4), and 5 mm (L5)). All electrodes have a 1 mm inter-loop distance (ILD).

**Figure 1:**
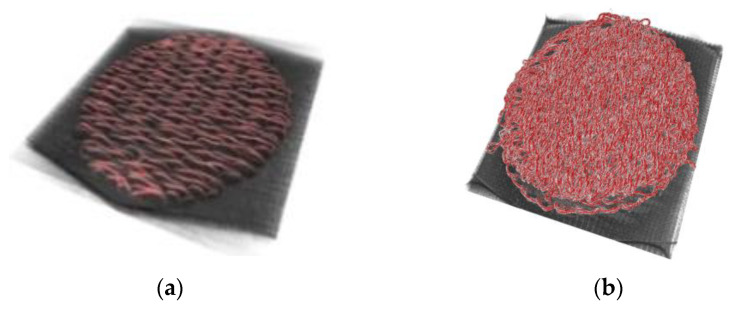

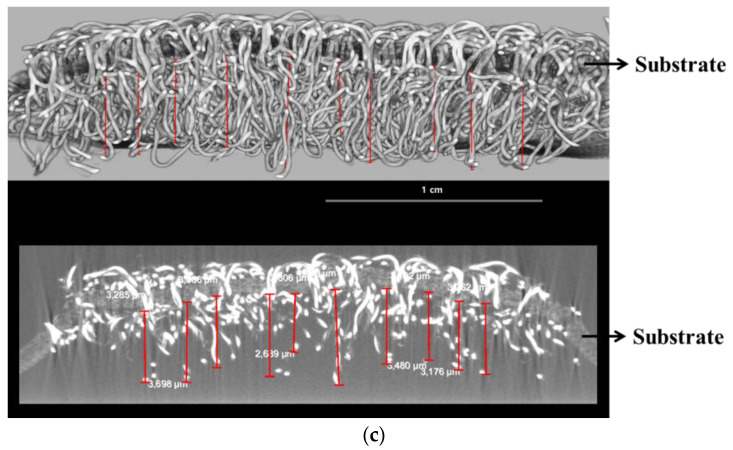
3D image of a (**a**) lock- and (**b**) moss-based embroidery textile electrode and (**c**) the loop height of the moss stitch [17].

Figure 1 shows 3D images of a moss electrode taken using X-ray microscopy [17]. As shown Figure 1a,b, the lock stitch is parallel to the base fabric and the moss stitch is created horizontal to the base fabric. Therefore, in moss stitch, a conductive area is formed in the form of a loop, and it can be seen that the conductive area increases in the vertical direction as the loop length increases.

### 2.3. Preparation for Leg Sleeves with Moss sEMG Electrodes

**Figure 2:**
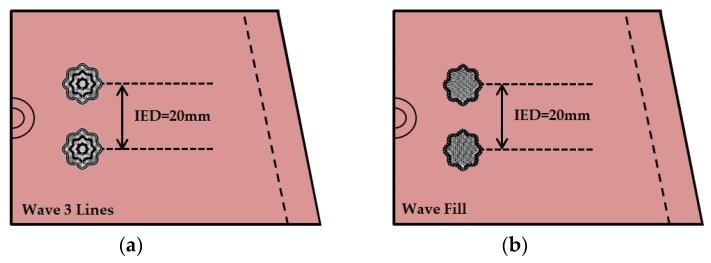
Embroidery design parameter for sEMG moss electrodes in different shapes: (**a**) wave three lines shape and (**b**) wave fill shape. IED = 20 mm; electrode size = 20 mm.

Figure 2 shows the embroidery design of the leg sleeve, which is an embedded moss electrode for sEMG with different shapes. The leg sleeves were fabricated to measure the clothing pressure and sEMG signal by applying the measurement position to the rectus femoris muscle. The effects of the loop size were examined by preparing five variations in loop length from 1–5 mm. According to a previous study, the inter-electrode distance (IED) was fixed at 40 mm, and the sleeve pattern applied a 30% pattern reduction rate (PRR), considering the elasticity of the substrate fabric [15].

**Figure 3:**
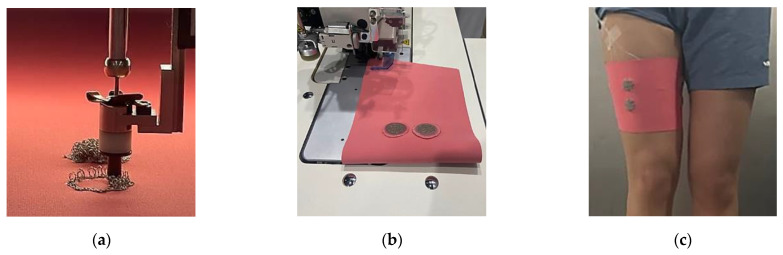
Procedure for preparing the leg sleeves with sEMG electrodes embroidered with a moss stitch. (**a**) Embroidery with a moss stitch technique, (**b**) sewing automatically with a 10 mm seam allowance, and (**c**) leg sleeves embroidered with a moss sEMG electrode.

Figure 3 presents the process of manufacturing leg sleeves in which moss sEMG electrodes are embedded. As shown in Figure 3a, electrodes are embroidered on a wide fabric of 120 cm × 180 cm (IED = 20 mm). The fabric-embroidered moss electrode was made into leg sleeves with an automatic sewing machine (AE-200A, JUKI, Tokyo, Japan), as shown in Figure 3b. Figure 3c presents the final manufactured leg sleeve. This study was performed on the same subject as in a previous study [16] (sex = female, age = 33 years, height = 175 cm, body weight = 60 kg). This study was approved by the Institutional Review Board of the Korea Institute of Industrial Technology (KITECH) (IRB No. A-2022-003 22 June 2022).

### 2.4. Characterization

#### 2.4.1. Tactile Properties of Moss Electrodes

Tactile comfort is attributed to the mechanical and structural properties of the fabric. Among the factors, the surface roughness and contact coolness were selected and evaluated. The tactile comfort of the electrode according to the loop length was compared with tactile comfort, which is the degree of comfort when in contact with the skin. Moss electrodes with loop lengths of 1 mm (WF_L1), 3 mm (WF_L3), and 5 mm (WF_L5), respectively, were selected and evaluated.

The surface roughness was measured using the Kawabata Evaluation System (KES-FB4-A, KATO TECH CO., LTD., Japan) according to KS K ISO 12947-4:1998, and the MIU (coefficient of fabric surface friction), MMD (mean deviation of MIU), and SMD (geometrical roughness, μm) values were calculated and analyzed. The contact coolness and warmth were compared with the Q-max measured using a Thermofeel (PF-QMM-01, Profid Co., Ltd., Japan) according to JIS L 1927:2020. The measurement environment temperature and humidity were 20 ± 2 °C and 65 ± 4% R.H., respectively.

#### 2.4.2. Clothing Pressure of the Moss sEMG Electrodes

**Figure 4:**
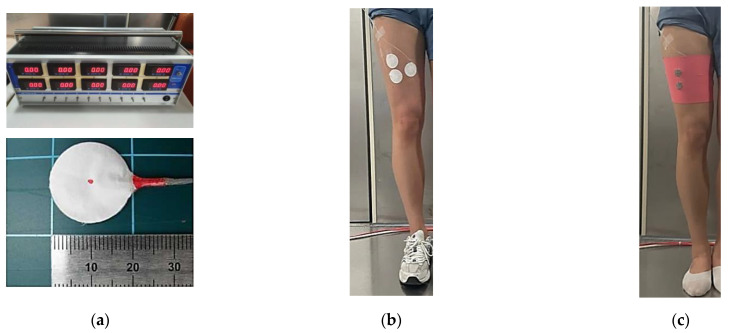
Clothing pressure measurement with leg sleeves embroidered with moss electrodes. (**a**) Pressure measurement machine and sensor with 20 mm diameter; (**b**) attached pressure sensor on to rectus femoris; (**c**) worn leg sleeves embroidered with moss electrodes.

In the same leg sleeve pattern, the loop length of the moss-type electrode was different, and the clothing pressures of the electrode and non-electrode parts were compared. The clothing pressure of the moss-type electrodes was measured using a TNB-300 (AMI Tech, Co., Ltd., Tokyo, Japan). A contact pressure sensor (AMI 3037-2, AMI Tech, Co., Ltd., Tokyo, Japan) was used to measure the clothing pressure imposed by the loop lengths of moss-type electrodes. The leg sleeve was prepared with a PRR (pattern reduction rate) of 30%, according to a previous study [16]. As shown in Figure 4, the pressure sensors were placed in three positions: two on the rectus femoris, where the electrode is located (electrode position), and the other on the other side of that muscle (non-electrode position). In addition, the leg was covered directly by the moss-type sEMG embroidered leg sleeves. The standing posture was maintained for one minute to obtain stable values.

#### 2.4.3. Electrical Properties of Moss sEMG Electrodes

The electrical properties of moss electrodes manufactured with different shapes and various loop lengths were evaluated through sheet resistance and skin-electrode impedance analysis. The sheet resistance of each fabricated electrode was measured in a laboratory environment using a four-point probe conductivity meter (RSD-IG 4-Probe, DASOLENG., Chengju, Republic of Korea). The skin-electrode impedance of the moss sEMG electrodes was measured in a laboratory environment by wearing a leg sleeve and using ZIVE SP2 ELECTROCHEMICAL WORKSTATION. The impedance corresponding to 1 to 1000 kHz was measured, and the impedance corresponding to 100 kHz was extracted and used.

#### 2.4.4. sEMG Measurement and Data Processing

The sEMG signal was evaluated by analyzing the characteristics of the EMG signals of various embroidery electrodes during knee extension. Bipolar EMG recordings were obtained using the embroidery electrodes on the rectus femoris, which is located on the anterior thigh and is involved in knee extension and hip flexion, according to a previous study [16]. No skin preparation (e.g., shaving and exfoliation) was performed before the test, considering that people would not implement skin preparation when wearing sEMG suits in practical use. Both embroidery electrodes were positioned on the midpoint of the muscle belly for the rectus femoris. Reference electrodes, pre-gelled self-adhesive Ag/AgCl electrodes (Kendall LTP, Covidien, MA, USA), were placed over the head of the fibula, an electrically neutral bony prominence. The recordings were amplified and filtered (20–500 Hz) in analog (MP160, BIOPAC Systems Inc., Goleta, CA, USA). The data were full-wave rectified and averaged with a 100 ms time constant to draw the amplitude of the signals. The entire data processing of the sEMG was performed using AcqKnowledge 5.0.1 Software (BIOPAC Systems Inc., Goleta, CA, USA) [16,17].

The baseline EMG signals were collected in the supine position for 10 s to measure the baseline electrode noise before measuring muscle contraction. A single-joint knee extension was carried out to induce muscle contraction signals while the subject was seated. The subject extended the knee until the lower leg was parallel to the floor. The test consisted of five consecutive trials for knee extension and knee flexion. Each phase lasted for five seconds. Three contractions among five phases, except the first and last trials, were used to calculate the average activated sEMG. The average and standard deviation were calculated to identify the significance of the differences between three samples in an identical configuration. The whole experiment was performed for each electrode studied: W3 and WF electrodes with five different loop lengths. During the entire experimental procedure, the room temperature was 25 °C and the relative air humidity was 60%.

#### 2.4.5. Data Analysis and Statistics

Non-parametric statistics were used to verify the significance of between-group differences owing to insufficient sample size for parametric methods. The Kruskal-Wallis test was used to identify group differences with the Mann-Whitney U test was used as a post hoc test. Statistical significance was reported at a level of *p* < 0.05.

## 3. Results and Discussion

### 3.1. Tactile Properties of Moss sEMG Electrodes

The MIU, which is the surface friction characteristic, MMD, which is the average deviation, and SMD, which represents surface roughness due to geometrical irregularities, were measured to compare the tactile comfort of the moss electrode. Among them, SMD represents geometric roughness; the larger the value, the rougher the surface is [19]. In general, this test measures two directions, weft and warp, respectively. On the other hand, the values according to warp and weft will be discussed as the average value measured five times each because the moss stitch has no significant effect on the direction in the form of a loop.

**Figure 5:**
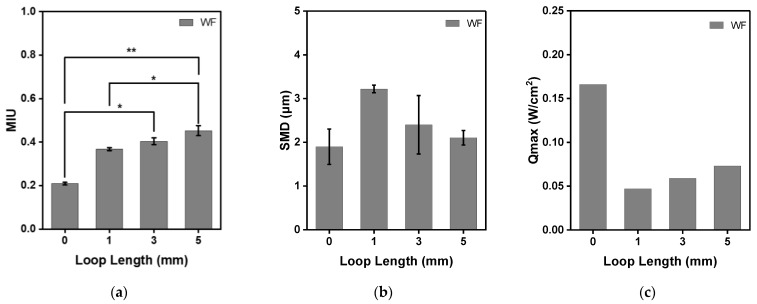
KES-FB4 surface testing results and Qmax of moss sEMG electrodes with various loop lengths. (**a**) MIU; (**b**) SMD; (**c**) Qmax, where loop length 0 represents the base fabric. * *p* < 0.05, ** *p* < 0.01.

As shown in Figure 5, the surface friction values of the base fabric, WF_L1, WF_L3, and WF_L5, were 0.209 ± 0.011, 0.368 ± 0.015, 0.404 ± 0.013, and 0.453 ± 0.015, respectively. The MIU values of the three moss electrodes were larger than that of the base fabric, and it was confirmed that they increased in proportion to the loop length (*p* = 0.016, Figure 5a). Electrodes with 1 mm and 5 mm loop lengths showed significant differences compared to base fabric (*p* < 0.05 for L = 1 mm and *p* < 0.01 for L = 5 mm). On the other hand, the SMD values representing geometric roughness were slightly different. The SMD values of the base fabric, WF_L1, WF_L3, and WF_L5, were 1.899 ± 0.404, 3.220 ± 0.088, 2.400 ± 0.667, and 2.104 ± 0.166μm, respectively. The SMD value of the moss electrode was larger than that of the base fabric, but the loop lengthened. Qmax represents the instantaneous contact cold-and-hot sensation, which is the value of heat transfer at the moment the skin touches the surface of the sample. Therefore, a large Qmax value can be interpreted to mean that heat transfer is large and cold sensation is large. The Qmax values of the base fabric, WF_L1, WF_L3, and WF_L5, were 0.166, 0.047, 0.059, 0.073 W/cm^2^, respectively. Therefore, the moss stitch has a warmer feel than base fabrics, and as the stitch length becomes longer, it slightly changes to a cooler feel.

### 3.2. Clothing Pressure of Moss sEMG Electrodes

**Figure 6:**
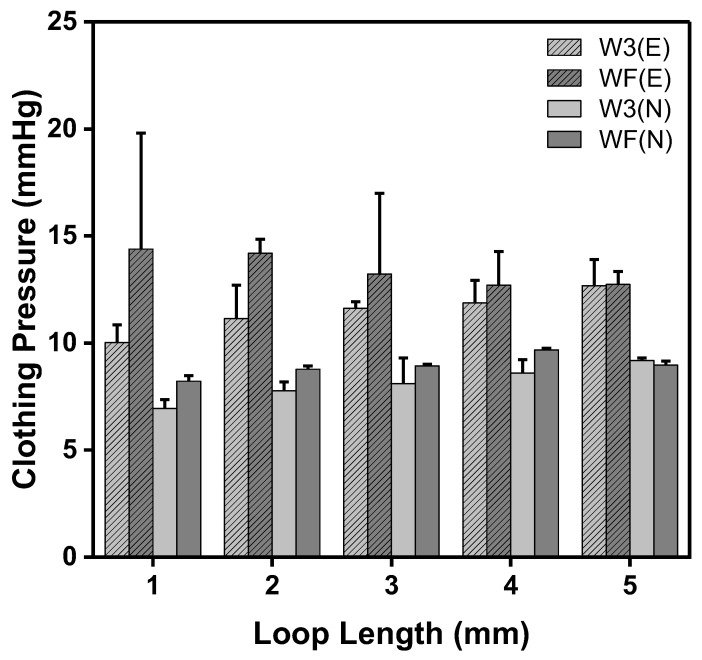
Partial clothing pressure results of the W3 and WF shape moss sEMG electrodes with various loop lengths. W3(E) and WF(E) are the electrodes at the rectus femoris position and non-electrode position, respectively.

The clothing pressure was measured at two partial pressures. The electrode position (E) refers to the area where electrode that measured sEMG was placed on the rectus femoris, and the non-electrode position (N) was the area without an electrode. Figure 6 presents the clothing pressure of moss sEMG electrodes with various shapes and loop lengths. The range of the clothing pressure corresponded approximately to the range that Kim and Lee [20] reported in their investigation of the clothing pressure of commercial compression sportswear, which was found to be 5.0–13.7 mmHg. In all electrodes, the clothing pressure on the electrode position was higher than that on the non-electrode position. This is because of the loops of moss sEMG electrodes protruding on the surface of the base fabric, and it appears that the more loops there are, the better the contact between the electrode and the skin.

The clothing pressure of the W3 electrode tended to increase as the loop length increased at the electrode position, and the clothing pressure was the highest for the electrode with the longest loop length (5 mm), at 12.68 ± 1.23 mmHg (Figure 6, W3(E)). On the other hand, the clothing pressure of the WF electrode showed a different tendency from the W3 electrode. The clothing pressure of the WF electrodes decreased as the loop length increased, and the highest clothing pressure was 14.38 ± 5.42 mmHg in the WF with a loop length of 1 mm (Figure 6, WF(E)). The clothing pressure at the non-electrode position of the W3 and WF electrodes was measured in the range of 6.93 to 9.19 mmHg. As shown in Table 1, the moss sEMG electrode has a non-conductive area (without a conductive thread), which lowers the clothing pressure. The volume of non-conductive area and shorter loop length were observed more in the W3 electrode. As the loop became longer, it was folded and placed in the surrounding non-conductive area because of the clothing pressure of the leg sleeve. The clothing pressure increased as the loop length increased in the case of the W3 electrode, with many non-conductive areas, because the electrode area was filled. On the other hand, the WF electrode with small non-conductive area swells and a cushion-like shape decreased the clothing pressure.

### 3.3. Electrical Properties of the Moss sEMG Electrodes

**Figure 7:**
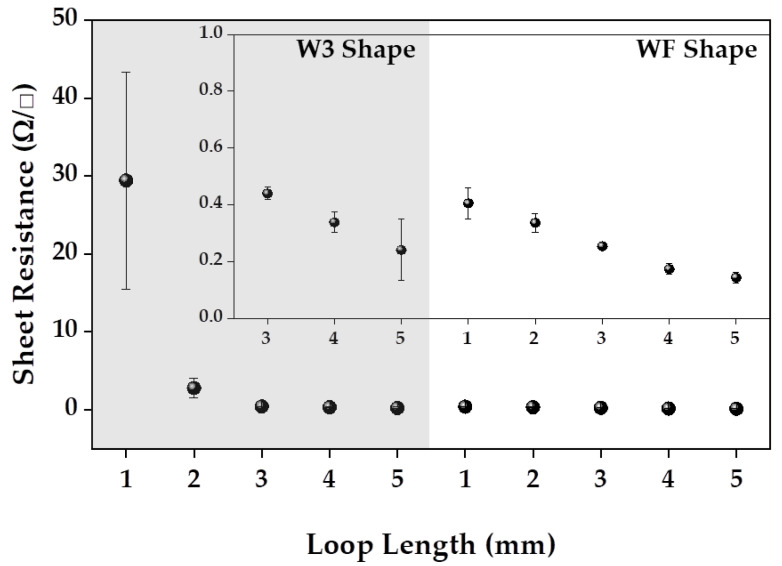
Sheet resistance of moss sEMG electrodes with various shapes (W3 and WF) and loop lengths (L = 1 mm, 2 mm, 3 mm, 4 mm, and 5 mm).

Figure 7 presents the sheet resistance to confirm the electrical characteristics according to the shape and loop length results of the embroidered moss sEMG electrodes. The sheet resistance was in the order of WF_L5 < WF_L4 < W3_L5 < WF_L3 < W3_L4, WF_L2 < W3_L3 < WF_L1 < W3_L2 < W3_L1. The sheet resistance of the electrodes of the same shape increased as the loop length increased, and in the case of the electrodes with the same loop length, W3 > WF. Among the 10 electrodes, W3_L1 had the highest sheet resistance of 29.44 ± 13.95 Ω/**□**, and WF_L5 had the lowest sheet resistance of 0.14 ± 0.02 Ω/**□**.

The sheet resistance can be expressed as the product of the resistance and the proportional constant, *K_S_*, and the following equation.

*R_S_* =
*K_S_* · *R* (1)

*K_S_* is a proportional constant defined as Equation (2).

*K_S_* =
*F*(*D*/*S*) · *F*(*t*/*S*) · *F*(*T*) · *F*(*S*) (2)

Here, *F*(*D/S*), *F*(*t/S*), *F*(*T*), and *F*(*S*) are correction coefficients and are values related to sample size, sample thickness, measurement temperature, and probe interval, respectively. In this study, only the correction factor for the thickness was considered for the sheet resistance. This is because the moss sEMG electrode sizes (20 mm), resistance measurement temperatures (room temperature), and probe spacing (4 μm) were the same.

According to Pouillet’s law [21], resistance can be described as the ratio of the length and cross-sectional area of a conductor, which is expressed as Equation (3):

(3) R=ρ·(l/A)

where *R*, ρ, l, and *A* are the resistance of the conductor, resistivity of the conductor, length of the conductor, and area of the conductor, respectively. The resistivity (ρ) is a unique value depending on the material of the conductor, and this value is equal because the same conductive thread was used in this study. The l value, the length of the conductor, was the same as the diameter of the electrode, 20 mm. Therefore, the resistance of the same loop-length electrode varies according to the cross-sectional area. Therefore, the sheet resistance is inversely proportional to the area (*A*) of the electrode, and the loop length (*L*) and can be expressed as Equation (4).

(4) $$R_{S}\propto \;(1/A)\cdot \;F(t/S)\propto \frac{1}{A\times L}$$

As shown in Table 1, the WF shape has a larger area (*A*) of conductive thread than the W3 shape at the same loop length. Therefore, the sheet resistance of the WF shape with a large conductive yarn area is stable and smaller than that of W3. A thicker sample means a smaller *F*(*t/S*) value [22,23]. Therefore, the sheet resistance decreases as the sample thickness increases, according to Equation (2). In this study, the loop length was considered as the thickness of the electrode, and the sheet resistance decreased as the loop length increased in both the W3 and WF shapes. On the other hand, electrodes with a loop length longer than 3 mm were less stable. According to Equation (4), the sheet resistance decreases as the loop length of the electrode becomes longer. As shown in Table 1, the non-conductive area of the electrode was reduced significantly when the loop length was more than 3 mm. This reduction means that the electrical stability of the electrode is improved because contact can be maintained between near loops.

The subject wore a leg sleeve embedded with electrodes, which each had a different shape and loop length for measuring skin–electrode impedance. Generally, higher skin–electrode impedance reduces common-mode rejection by increasing the impedance mismatch, thus reducing the signal-to-noise ratio (SNR) [24]. Therefore, the low and stable impedance of the electrode is significant for the high-fidelity acquisition of bio-signals.

**Figure 8:**
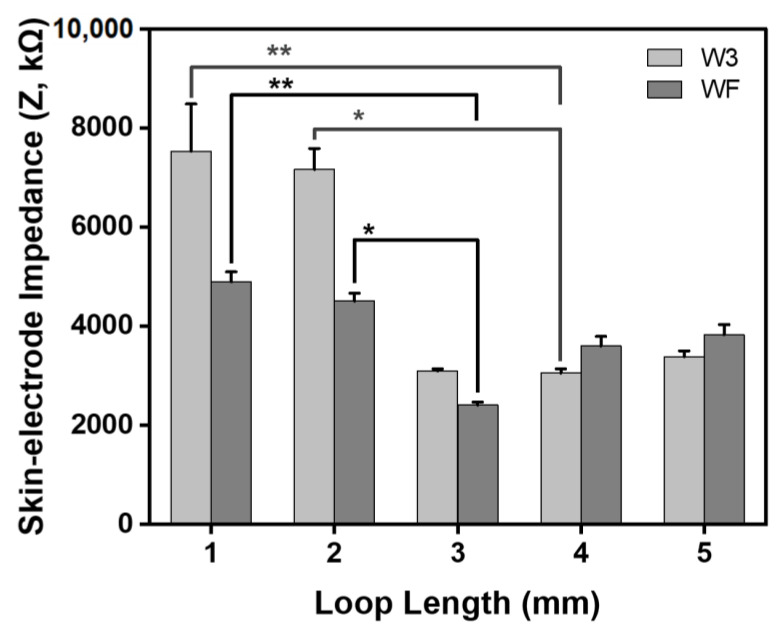
Skin–electrode impedance of moss sEMG electrodes for two shapes (W3 and WF) and various loop lengths (L = 1 mm, 2 mm, 3 mm, 4 mm, and 5 mm). * *p* < 0.05, ** *p* < 0.01.

Figure 8 shows the skin–electrode impedance at 100 kHz in moss sEMG electrodes of different shapes and various loop lengths. The impedance according to the electrode shape showed a smaller value for the WF electrodes than the W3 electrodes, similar to the sheet resistance results.

In the case of the W3 electrodes, the impedance values of W3_L1, W3_L2, W3_L3, W3_L4, and W3_L5 were 7.532 ± 0.95 kΩ, 7.163 ± 0.421 kΩ, 3.099 ± 0.039 kΩ, 3.057 ± 0.083 kΩ, and 3.386 ± 0.117 kΩ, respectively. The impedance significantly decreased as the loop length increased; among the results, the value of W3_L4 was the smallest (*p* = 0.015, Figure 8). The values were 59% and 57% smaller than values of W3_L1 (** *p* < 0.01) and W3_L2 (* *p* < 0.05), respectively. On the other hand, the impedance was saturated slightly when the loop length was longer than 4 mm.

In the case of the WF electrode, the impedances of WF_L1, WF_L2, WF_L3, WF_L4, and WF_L5 were 4.896 ± 0.199 kΩ, 4.504 ± 0.161 kΩ, 2.410 ± 0.059 kΩ, 3.600 ± 0.193 kΩ, and 3.830 ± 0.203 kΩ, respectively. Impedance was the smallest for the WF_L3 electrode, being 51% and 47% smaller than values of WF_L1 (** *p* < 0.01) and WF_L2 (* *p* < 0.05), respectively. Similar to the results of the W3 electrode, the impedance showed different changes before and after reaching loop lengths of 3 mm (* *p* < 0.05).

The degree of change in impedance due to changes in loop length was larger for the W3 electrode when the loop length was less than 3 mm and larger for the WF electrode when the loop length was larger than 3 mm. Therefore, as discussed above, the clothing pressure increases when the loop length is long, but if the loop is too long, the skin–electrode impedance increases by reducing the skin adhesion of the electrode.

### 3.4. sEMG of the Raw Signal and Average Rectification of the Moss sEMG Electrodes

The performance of the moss electrodes in detecting sEMG signals was validated by analyzing the sEMG signal characteristics with two variables (electrode shape and loop length) during knee extension. Based on a previous study, the sEMG signal of the rectus femoris was obtained and evaluated by repeating the knee flexion–extension process five times while putting on a leg sleeve with moss sEMG electrodes embroidered. The sEMG signal was obtained from each electrode by repeatedly measuring five seconds of rest periods after each flexion. Three contractions among five trials, except the first and last trials, were used to calculate the average activated sEMG for comparison [18].

**Figure 9:**
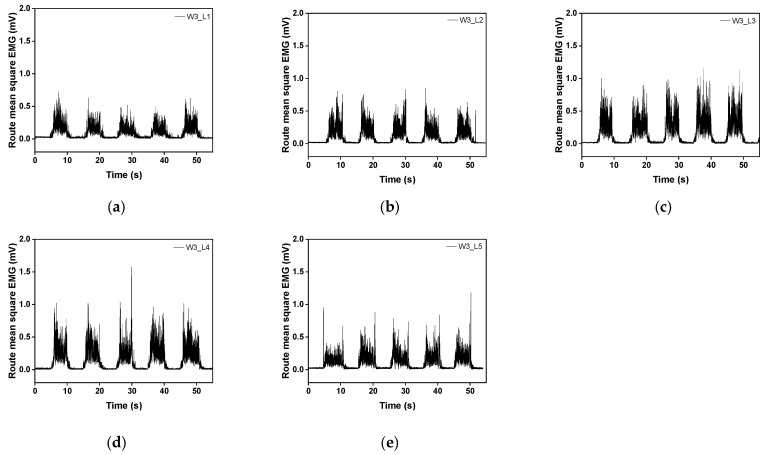
Graph of filtered (20-500 Hz) in analog and full-wave of the sEMG signal obtained by (**a**) W3_L1, (**b**) W3_L2, (**c**) W3_L3, (**d**) W3_L4, and (**e**) W3_L5. Here, the values of sections A, B, and C in the box were used for EMG signal measurement and analysis.

**Figure 10:**
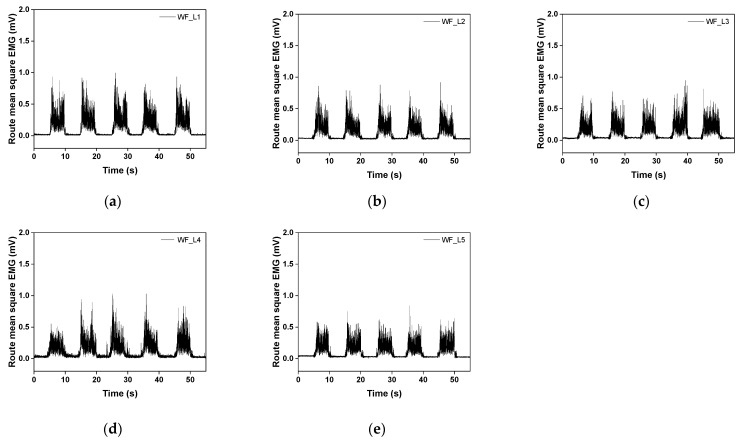
Graph of filtered (20-500 Hz) in analog and full-wave of the sEMG signal obtained by (**a**) WF_L1, (**b**) WF_L2, (**c**) WF_L3, (**d**) WF_L4, and (**e**) WF_L5. Three contractions among the five trials, excepting the first and last trials, were used to calculate the average activated EMG for comparison.

Figure 9 and Figure 10 show the raw and filtered rectified sEMG values of the embroidery-based textile electrodes. All moss sEMG electrodes could detect the sEMG signal. In addition, the magnitude and stability of the sEMG signal could change according to the shape and loop length of the electrode. The raw signal of W3 tended to increase as the loop length increased from L1 to L4 (Figure 9). On the other hand, the signal value tended to decrease after W3_L4. In addition, the magnitude of the sEMG signal detected with the WF-type electrodes was slightly larger than with the W3-type electrodes (Figure 10). In the case of WF, a similar signal size was shown regardless of the loop length. The signal size varied depending on the shape and length of the loop. W3 has a relatively low density, while WF has the entire area embroidered with conductive yarn. Accordingly, as the loop length increases from W3_L3 and W3_L4, the empty part is filled to maintain a shape that matches the location and size of the muscle to be detected. After L4, the muscle size exceeded the signal size and caused noise.

**Figure 11:**
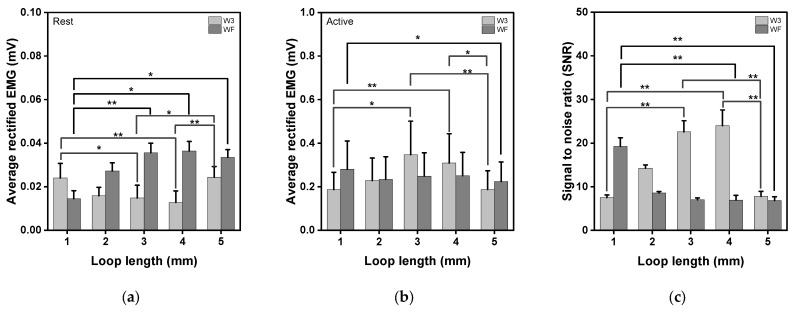
Average rectified EMG at the baseline and during knee extension for moss sEMG electrodes of various shapes and loop lengths. (**a**) Baseline electrode noise, (**b**) activated muscle signal during knee extension, and (**c**) signal-to-noise ratio (SNR). * *p* < 0.05, ** *p* < 0.01.

Figure 11 presents the average rectified sEMG of the baseline before and after muscle activation and the signal-to-noise ratio (SNR) for the various electrode types. sEMG at the baseline is the noise of the electrode, as shown in Figure 10a. A smaller value means a more stable electrode, and a higher activated sEMG value means better collecting performance. The SNR was calculated to activate the sEMG signal compared to the background electrode noise.

During knee extensions, SNR consistently and significantly increased with the increasing loop length (*p* = 0.002, Figure 11a). The baseline electrode noise range was from 0.01 mV to 0.04 mV, showing similar values for different electrode shapes and loop lengths. As the loop length increased, the noise value decreased for the W3 moss sEMG electrode, whereas it gradually increased for the WF moss sEMG electrode (*p* = 0.004 and *p* = 0.005).

Among the results of activated muscle signal testing, W3_L3 and W3_L4 electrodes showed higher values than others (Figure 11b). As the loop length increased, the sEMG value increased for the W3 moss sEMG electrode, whereas it slightly decreased for the WF moss sEMG electrode (*p* = 0.004 and *p* = 0.039). As explained previously, the W3 electrode had a hollow interior compared to the WF electrode. The empty space of the electrode can be filled with surrounding elongated loops, improving the performance of the electrode. However, when the loop length reaches 5 mm, there is no more space to fill, which may exceed the electrode diameter (20 mm) and reduce signal performance.

## 4. Conclusions

Technical embroidery techniques were used to manufacture textile-type electrodes for acquiring biological signals. The conditions for electrodes optimized for wearable smart clothing were derived using a moss stitch designed according to the shape and length of the loop and analyzing the tactile comfort, clothing pressure, electrical property, skin-electrode impedance, and sEMG performance.

The tactile comfort results showed that the surface unevenness increases as the loop length increases, but the surface roughness tends to decrease due to the free movement of the thread constituting the loop. An analysis of the clothing pressure according to the shape and loop of the electrode manufactured with a moss stitch revealed it to be similar to the clothing pressure value of commercial compression sportswear, at 5.0–13.7 mmHg. W3 showed a tendency for the clothing pressure to increase as the loop length increased, filling the void. On the other hand, WF had a cushion-like shape as the loop length increased, reducing the clothing pressure. The electrical properties of the electrode were confirmed through sheet resistance measurements, showing that, regardless of the shape, the contact points between conductive strands of yarn increased as the length of the loop increased, while the sheet resistance value tended to decrease. On the other hand, the skin–electrode impedance showed different results, showing a tendency to decrease up to 3 mm and increase as it went to 5 mm. This is because the loop filled the empty space within the electrode when the electrode contacted the skin. In addition, a longer loop reduced skin adhesion and increased the skin–electrode impedance. Moreover, the sEMG performance showed excellent SNR values in WF_L3 and WF_L4 because W3, which has a lower density and empty spaces compared to WF, can fill the empty spaces as the length of the loop increases.

Therefore, the moss stitch-based textile-type embroidery electrode developed in this study showed improved wearability with excellent bio-signal collection performance and can be applied as an electrode in various wearable clothing for collecting bio-signals in the future.

## Figures and Tables

**Table 1:** Sample codes and embroidery conditions for manufacturing moss sEMG electrodes.

Sample Code	W3_L1	W3_L2	W3_L3	W3_L4	W3_L5	WF_L1	WF_L2	WF_L3	WF_L4	WF_L5
Designed Image	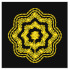					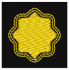				
Real Image *	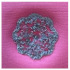	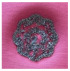	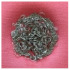	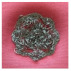	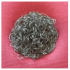	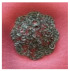	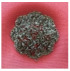	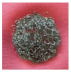	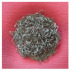	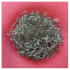
Loop Length (mm)	1	2	3	4	5	1	2	3	4	5
Shape	Wave 3 Lines					Wave Fill				
Electrode Diameter (mm)	20									
ILD (mm)	1									

* Real Image: Top view.

## Data Availability

The data presented in this study are available in this article.
